# Validity of the Parsley Symptom Index—an Electronic Patient-Reported Outcomes Measure Designed for Telehealth: Prospective Cohort Study

**DOI:** 10.2196/40063

**Published:** 2022-11-03

**Authors:** Hants Williams, Sarah Steinberg, Kendall Leon, Catherine O’Shea, Robin Berzin, Heather Hagg

**Affiliations:** 1 Department of Applied Health Informatics School of Health Professions Stony Brook University Stony Brook, NY United States; 2 Parsley Health New York, NY United States

**Keywords:** telemedicine, eHealth, mHealth, web-based

## Abstract

**Background:**

Electronic patient-reported outcomes measures (e-PROMs) are a valuable tool for the monitoring and management of chronic conditions over time. However, there are few validated tools available that capture symptoms across body systems in telehealth settings. The Parsley Symptom Index (PSI) is a recently developed symptom assessment for adults with chronic disease in telehealth settings. A previous study demonstrated the feasibility and acceptability of the PSI in a clinical telehealth setting.

**Objective:**

The purpose of this study was to assess convergent validity between the PSI and the self-rated health (SRH) item.

**Methods:**

This prospective cohort study took place from January 15, 2021, to December 15, 2021, among a sample of 10,519 adult patients at Parsley Health, a subscription-based holistic medical practice. The PSI and the SRH were completed by patients via an online portal. The association between the PSI and SRH was assessed via polyserial and polychoric correlations, while weighted κ scores provided information related to agreement between the PSI and SRH.

**Results:**

From 22,748 responses, there were moderate levels of association (polyserial *r*=0.51; polychoric *r*=0.52) and agreement (weighted κ=0.46) between the PSI and SRH. In total, 74.13% (n=16,865) of responses between the PSI and SRH were relatively congruent while 36.17% (n=8229) were literally congruent.

**Conclusions:**

The PSI demonstrates convergent validity with the SRH for adults with chronic disease in a telehealth setting. This finding further supports the validation of the PSI in a real-world clinical setting. Although it is conceptually similar to the 1-question SRH, the PSI is a 45-item PROM designed to capture quality of life and specific symptoms by body system. Future studies will compare the PSI to multi-item PROMs.

## Introduction

Providing telehealth options has become indispensable to health care delivery in the United States. Even before the COVID-19 pandemic fundamentally altered the health care landscape, claim lines in the United States for nonhospital-based clinicians to patient telehealth grew 1393% [1] from 2014 to 2018.^.^ Health crisis triaging during the COVID-19 pandemic further increased demand for telehealth care [2,3], accelerating the transition from brick-and-mortar practice to the virtual interface. The pandemic spawned an entirely new telehealth industry, reducing access and cost barriers for patients, from the rural farmer to the busy urban professional [4,5].

Having access to affordable care is especially important for the 60% of Americans that live with at least 1 chronic disease, and this group spends 2 to 4 times more on health care than do those without any chronic conditions [6]. Telehealth helps clinicians effectively manage chronic disease with increased opportunity to monitor treatments and quickly respond to patient concerns [7], which reduces costs [8] and hospitalizations [9]. Electronic patient-reported outcome measurements (e-PROMs) are tools that serve as the first patient touchpoint in a telehealth consultation generally and in particular can play a pivotal role in the clinical care of patients with chronic conditions. Completing e-PROMs allows patients to reflect on their own health, boosts patient-clinician communication, and empowers patients to steer their own health care journey [10].

Despite the fact that many Americans with chronic diseases are currently being treated via telehealth, there are limited e-PROM tools available to telehealth providers and clinics for assessing and tracking a patient’s health status over time. Tools like the Patient-Reported Outcomes Measurement Information System (PROMIS) [11,12], the 36-Item Short Form Health Survey (SF-36) [13,14], and the Medical Symptom Toxicity Questionnaire (MSQ) [15] are powerful e-PROM tools for tracking a patient’s health status over time, but none of them offer a single, short-form assessment that could be easily integrated into the clinician workflow or electronic medical record or that can capture symptoms across body systems like a review of systems (ROS).

As part of a larger effort to leverage new tools like e-PROMs to make the telehealth experience engaging and effective for patients with chronic diseases, a research team at Parsley Health (a subscription-based holistic medicine practice) built the Parsley Symptom Index (PSI). The PSI is a 45-item e-PROM designed specifically for use in telehealth settings to function as an ROS. When used strategically, a patient-reported outcome–driven approach can shift an ROS to a cooperative dialogue between patients and clinicians [16]. Like an ROS, the PSI focuses on bodily domains and the most commonly reported symptoms associated with chronic conditions for each domain. As a digital-first e-PROM, we built the PSI to provide immediate feedback to patients, producing data that are seamlessly adopted into the standard clinical workflow and providing the scaffold for an effective patient-clinician conversation [17]. To our knowledge, the PSI is the only existing short form e-PROM developed with preliminary validation for use within a telehealth setting for patients with chronic disease [18].

In an initial feasibility and acceptability study that assessed construct and face validity, the PSI was deployed, completed, and found helpful to both patients and clinicians [18]. Having previously described the item generation, accessibility, and interpretability in a population receiving longitudinal care, we conducted this study is to continue validation of the PSI by comparing it against the self-rated health (SRH) score, a single-item question that has been successfully used in prior research to test construct validity of patient-perceived health [19-21].

## Methods

### Study Design

This prospective cohort study took place at Parsley Health from January 15, 2021, to December 15, 2021, among a sample of 10,519 adult patients. Patients completed the PSI and the SRH via an online portal. The average monthly PSI completion rate was 77.21% (range 69.23%-83.44%) over the study period.

**Ethics Approval**

This study used patient-reported survey data that were recorded in such a manner that participants were unidentifiable to the researchers. The institutional review board at Stony Brook University considered this study exempt (IRB2020-00429) from Code of Federal Regulations Title 45 requirements [22].

### Study Setting and Population

Parsley Health is a subscription-based membership model for delivering primary care and proactive chronic disease management through a holistic-medicine lens. Patients receive care from Parsley Health clinicians and health coaches in-person and remotely, with additional access to their care team via email and an online portal. Prior to the COVID-19 pandemic, Parsley Health further increased their telehealth availability to over 45 states. Inclusion criteria for this study were Parsley Health patients that had an active subscription membership plan between January 15, 2021, and December 15, 2021, and a minimum of 1 clinical encounter within their membership period. Exclusion criteria were severe psychiatric disorders (particularly psychosis and depression requiring a change in treatment in the last 30 days), age under 18 years, and being unable to speak or read English.

### Parsley Symptom Index

The PSI is a 45-item, ROS-style PROM tool designed to capture chronic disease symptoms [18]. The PSI development followed the framework outlined by the Federal Drug Agency (FDA) guide for PROM development [23]. Items are grouped into 9 systems, with each containing 4 to 7 items per group that are ranked on a scale from 0 (asymptomatic) to 10 (extremely symptomatic). A total score is calculated with the following 4 cutoff ranges: 0-24, 25-43, 44-71, and greater than 71. The respective terminology for these ranges are “well” (0-24), “symptomatic” (25-43), “very symptomatic” (44-71), and “sick” (71+). Upon completing the PSI, patients can immediately view their PSI score. When they meet with their clinician, they can view it in graphical format and compare it to past responses, stratified by body systems.

### Self-rated Health Item

The SRH item was administered alongside the PSI. The SRH was a mandatory item at the end of the PSI, and only complete questionnaires were included in this study. The SRH is a single question, with a 5-item Likert scale answer that reads as follows: “In general, would you say that your health is excellent, very good, good, fair, or poor?” The SRH is validated and is commonly used to demonstrate construct validity of PROMs [19-21] and allows the clinician to perform a quick global assessment of patient-perceived well-being.

### Procedure

After patients scheduled a visit, they were instructed to log into an online patient portal and complete the PSI 24 to 48 hours before each clinical visit. Initial visits were rescheduled if all forms were not completed, but follow-up visits were not postponed for an incomplete PSI. For follow-up visits, patients who had not completed the PSI received an automated reminder 48 hours before the clinical visit. If the PSI was not completed after the automated prompt, another prompt was sent from the clinician or clinical operations coordinator.

When clinicians prepared for an online visit, they used a standardized note template within the electronic health record to pull the most recent PSI score into the visit note. The PSI design allowed for the results to be immediately usable: once a PSI was completed, patients received instant feedback, and clinicians could quickly import the data into the note to prepare for the patient visit. With the PSI template integrated into the beginning of the encounter note, clinicians were subtly prompted to use the PSI to discuss patient-reported symptoms and provide positive feedback to the patient for completing the PSI.

During the telehealth patient visit, the PSI score was used as a touchpoint for the patient-clinician discussion. As the PSI was previously completed, clinicians were able to ask targeted questions about symptoms and had more time to focus on burden and distribution of illness. The longitudinal PSI graph further deepened the provider’s ability to identify triggers and mediators that influenced disease trajectory over time. 

### Association Analysis

To test the hypothesis that the SRH item would correlate with the PSI, 2 measures of association were calculated. First, a polyserial correlation was performed on the raw continuous score of the PSI (range 0-500) with the ordinal SRH categories (excellent, very good, good, fair, poor). Next, the PSI’s responses were scored and translated into ordinal categories (1=great, 2=good, 3=average, 4=fair, 5=poor) to compare directly with the SRH categories and generate polychoric correlation coefficients [24]. This second analysis provided an alternative view for when the PSI is interpreted as ordinal instead of continuous.

### Agreement Analysis

To determine agreement, weighted κ (quadratic) scores incorporated information about the distance between the transformed ordinal PSI and SRH ratings: ratings that were 1 category apart counted as “less disagreement” than did a pair of ratings 2 categories apart. The weighted κ method partially contributes to responses that are “near” the rating category; for example, “Very good” and “Excellent” are categorically closer than are “excellent” and “poor.” To interpret the κ score, the following guidelines are used to suggest agreement [25-28]: 0=agreement equivalent to chance, 0.10-0.20=light agreement, 0.21-0.40=fair agreement, 0.41-0.60=moderate agreement, 0.61-0.80=substantial agreement, 0.81-0.99=near-perfect agreement, and 1.00=perfect agreement.

In addition, a binary interpretation of agreement results as “literally congruent” or “relatively congruent” was calculated. If the PSI and the SRH were an exact match (eg, both scored as “Very good” or both “Poor”) the congruence type was scored as literal, while if an individual’s responses to the PSI and SRH were not an exact match but consistent in terms of their position as either good (“Excellent,” “Very good,” “Good”) or bad health (“Fair,” “Poor”), the congruence type was scored as relative [29].

### Data Analysis Software

All analyses were carried out in SAS version 9.4 (SAS Institute) [30].

## Results

There were a total of 22,732 observations from 10,519 unique patients from January 15, 2021, to December 15, 2021. Only completed sociodemographic data for patients are represented in Table 1. Race and gender identity data are not complete for the entire sample and were added in late January 2020 for new members. Missing data for race and gender for members registered prior to January 2021 are still being retroactively collected by staff. Data describing race or ethnicity and gender identity refer to the segment of the population for which that data are complete (n=8042).

The distribution of responses for each scale item was skewed toward the positive (Table 2). Of the 22,748 respondents, 12.45% (n=2834) and 3.58% (n=817) reported their health as “Excellent” for the PSI and SRH, respectively; 22.85% (n=5207) and 38.65% (n=8794) rated their health as “Very good” or “Good” for the PSI, respectively, and 25.31% (n=5759) and 42.79% (n=9734) as “Very good” or “Good” for the SRH, respectively. Fewer than 25.92% (n=5897) rated their health to be “Fair” or “Poor” on the PSI and 28.23% (n=6422) did so for the SRH.

The polyserial correlation between raw PSI scores and the SRH was *r*=0.51, suggesting moderate association. When the PSI scores were treated as ordinal (transformed to SRH scale), the polychoric correlation coefficient was nearly identical at *r*=0.52, also suggesting moderate association. The weighted κ coefficient between the transformed PSI and SRH was 0.46, suggesting moderate agreement (Table 3). The agreement analysis shows approximately 74.13% (16,865/22,748) relative congruence and 36.17% (8229/22,748) literal congruence across all observations.

**Figure 1:**
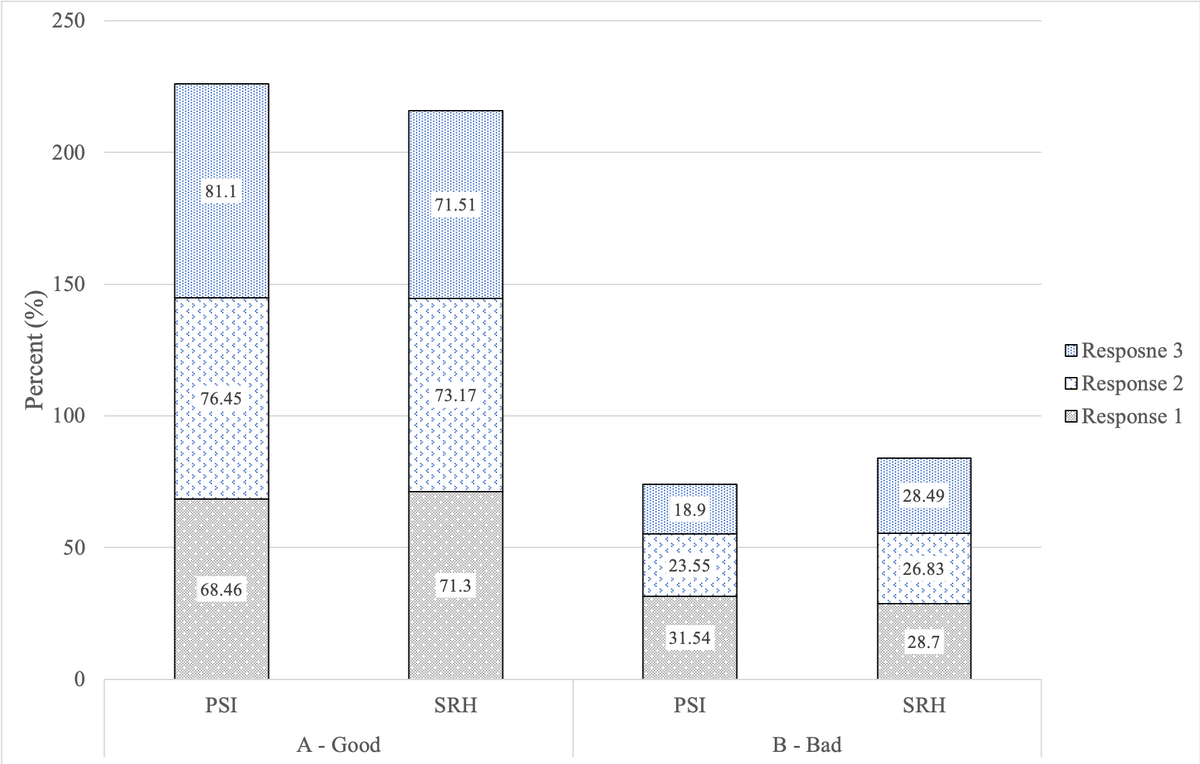
PSI by SRH responses across time: two categories. PSI: Parsley Symptom Index; SRH: self-rated health.

**Figure 2:**
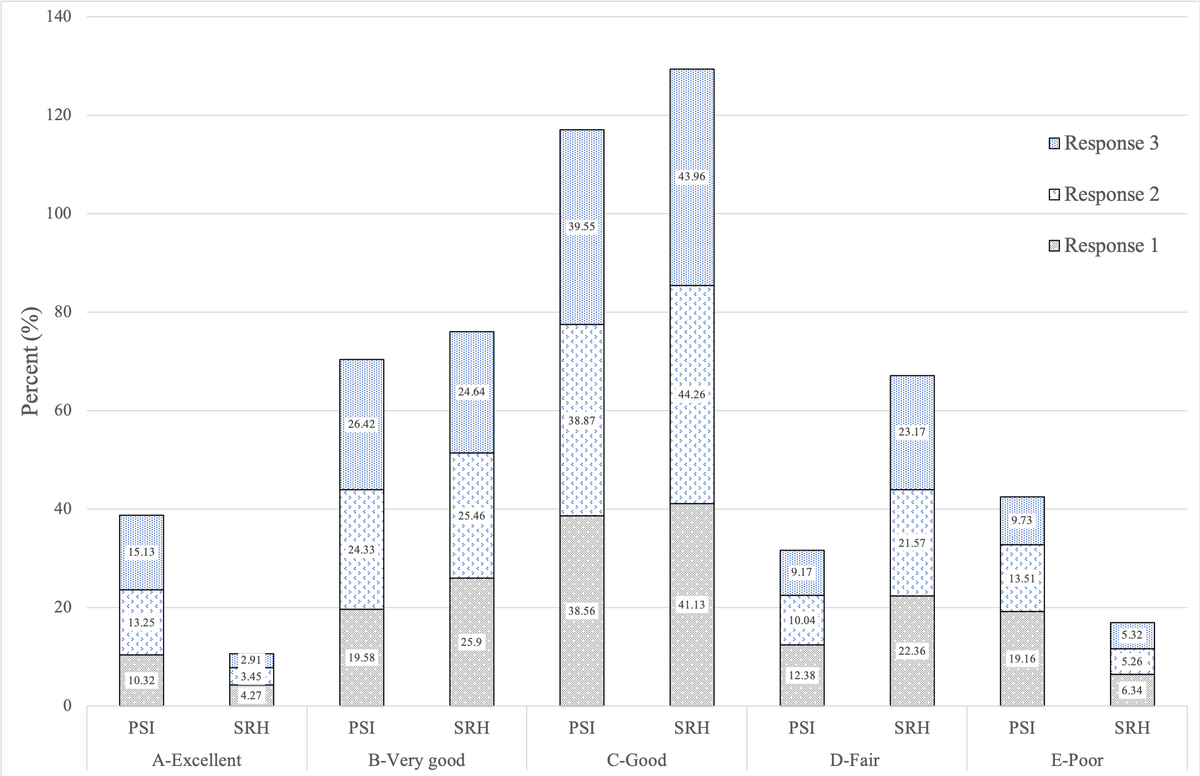
PSI by SRH responses across time: five categories. PSI: Parsley Symptom Index; SRH: self-rated health.

Although sample size diminishes with increasing visits, concordance between the PSI and SRH remains stable, even in the cells with smaller sample size. For graphic representation (Figure 1 and Figure 2), we limited our data to 1 to 3 visits for visual clarity. Lastly, in keeping with good reporting practices the Checklist for Reporting Results of Internet E-Surveys (CHERRIES) [31] is provided in Multimedia Appendix 1.

**Table 1:** Patient descriptives (N=10,531).

Characteristic		Value
**Biological Sex, n (%)**		
	Female	9092 (86.33)
	Male	1351 (12.82)
	Other	88 (0.83)
**Gender identity, n (%)^a^**		
	Woman	6942 (86.32)
	Man	1011 (12.57)
	Nonbinary	33 (0.41)
	Female to male	13 (0.16)
	Male to female	11 (0.13)
	Nonbinary other	13 (0.16)
	Transgender	6 (0.07)
	Gender queer	13 (0.16)
**Race, n (%)^a^**		
	White	6104 (75.90)
	American Indian or Alaskan Native	27 (0.33)
	Asian	508 (6.31)
	Black or African American	560 (6.96)
	Native Hawaiian or other Pacific Islander	23 (0.28)
	Other	690 (8.57)
	Prefer not to say	130 (1.61)
**Age group, n (%)**		
	18-24 years	459 (4.35)
	25-34 years	3931 (37.32)
	35-44 years	3346 (31.77)
	45-54 years	1604 (15.23)
	55-64 years	783 (7.43)
	65-74 years	326 (3.09)
	75-84 years	74 (0.70)
	85+ years	8 (0.07)
Number medical visits, mean (SD)		3.07 (3.11)
Number health coach visits, mean (SD)		2.30 (2.81)
**Total membership duration, n (%)**		
	0-1 year	7361 (69.89)
	1-2 years	1755 (16.66)
	3 or more years	1415 (13.43)
**Most frequent ICD^b^ codes, n (%)**		
	Abdominal distension (gaseous)	3300 (31.33)
	Other fatigue	3244 (30.80)
	Anxiety disorder, unspecified	2554 (24.25)
	Irritable bowel syndrome with diarrhea	1826 (17.33)
	Constipation, unspecified	1626 (15.44)
	Insomnia, unspecified	1105 (10.49)
	Hypothalamic dysfunction, not elsewhere classified	1079 (10.24)

^a^Due to missing data, N=8042 for this category.

^b^ICD: International Classification of Diseases and Related Health Problems.

**Table 2:** PSI^a^ and SRH^b^ descriptives (N=22,748).

Characteristic		Value, n (%)
**Total responses**		
	1 response	4333 (19.04)
	2 responses	5324 (23.40)
	3 responses	5823 (25.59)
	4 responses	3796 (16.68)
	5 responses	2345 (10.30)
	6 responses	786 (3.45)
	7 responses	224 (0.98)
	8 responses	80 (0.35)
	9 responses	27 (0.11)
	10 responses	10 (0.04)
**Concordance: relative**		
	Congruent	16865 (74.19)
	Incongruent	5883 (25.86)
**Concordance: literal**		
	Congruent	8229 (36.18)
	Incongruent	14519 (63.8)
**Time submitted**		
	Daytime	5655 (24.85)
	Evening	12146 (53.39)
	Morning	1172 (5.15)
	Night	3775 (16.59)
**PSI mapped to SRH categories**		
	Excellent	2835 (12.46)
	Very good	5209 (22.89)
	Good	8801 (38.68)
	Fair	2473 (10.85)
	Poor	3430 (15.05)
**SRH categories**		
	Excellent	817 (3.59)
	Very good	5761 (25.28)
	Good	9744 (42.76)
	Fair	5105 (22.40)
	Poor	1321 (5.79)

^a^PSI: Parsley Symptom Index.

^b^SRH: self-rated health.

**Table 3:** Association and agreement.

	Response count, n (%)	Polyserial correlation, *r*	Polychoric correlation, *r*	Relative concordance, n (%)	Literal concordance, n (%)	Weighted κ	Maximum κ
Total	22,732 (100)	0.517	0.522	16865 (74.19)	8229 (36.18)	0.460	0.754
Response 1	10,520 (46.28)	0.506	0.517	7623 (72.46)	3763 (35.77)	0.453	0.747
Response 2	6195 (27.25)	0.539	0.552	4704 (75.93)	2316 (37.38)	0.478	0.753
Response 3	3535 (15.55)	0.563	0.568	2674 (75.64)	1304 (36.89)	0.476	0.747
Response 4	1595 (7.02)	0.525	0.515	1190 (74.61)	535 (33.54)	0.421	0.715
Response 5	646 (2.84)	0.539	0.543	484 (74.92)	216 (33.44)	0.434	0.675
Response 6	177 (0.78)	0.665	0.660	138 (77.97)	65 (36.72)	0.545	0.664
Response 7	46 (0.20)	0.707	0.654	39 (84.78)	17 (36.96)	0.570	0.579
Response 8	14 (0.06)	0.687	0.569	10 (71.43)	6 (42.86)	0.446	0.897

## Discussion

### Principal Findings

This study investigated the concordant validity of the PSI, a digital-first e-PROM, by comparing it to the SRH in a large adult population. We found moderate association and agreement (ie, relative concordance) between the PSI and SRH. When the PSI was scored as an ordinal, it did not perfectly match the 5 health categories in the SRH; however, they were consistent in terms of their position as good health (excellent, very good, good) versus bad health (fair, poor). In other words, the PSI and SRH generally point in the same directions for self-reported health categorization.

Various analyses were performed to explore association and agreement. First, we analyzed whether collapsing PSI scores into ordinal categorical variables (vs continuous) would change the association with SRH. The results were similar between continuous (polyserial correlation) and categorical (polychoric correlation) when compared to the categorical SRH. *t* tests showed no significant difference between these correlations. We also explored whether agreement between PSI and SRH were different between the first patient visit versus subsequent visits. Agreement between PSI and SRH for patients with repeated assessments remained consistent over time, suggesting consistency for the PSI from first visit to follow-up visits.

We noted that patients tended to report better health on the PSI than on the SRH. In this study, the SRH question was asked at the end of the PSI. It is possible that while answering the PSI questions, patients were reminded of their health symptoms leading them to be more likely to rate their health poorly in the SRH. The order of administration may play a role in the agreement level [32]. Future studies should incorporate A/B testing to explore whether the order of administration impacts the self-reported perception of well-being.

Although the focus of this study was not to assess or describe longitudinal changes between the PSI and SRH, we did observe that the PSI captured improvement in symptoms over time with treatment (Figure 1 and Figure 2). In comparison, the SRH remained relatively static over time. This implies that the PSI, with its greater degree of granularity, can capture symptom changes in a way that we would not expect from a single-item question like the SRH [33]. We did observe a broad range of PSI and SRH responses that fell into a normal distribution, indicating the full spectrum of perceived health statuses. This normal distribution persisted over time for both measures, but the PSI as reported was more sensitive to detecting changes over time. Further research should investigate potential moderators and mediators that influence PSI response change over time, such as baseline health status, age, sex, race, and pre-existing conditions.

Beyond the effects of administration order, there are conceptual differences between the PSI and SRH that may contribute to the degree of agreement. Although they address the same broad clinical concepts, the 45-item PSI captures more information than does the single-item SRH [34]. We would expect a general trend of agreement or relative concordance between the two, but not to such a high degree that it would match perfectly (literal concordance).

The PSI was created because a short-form e-PROM to capture a review of systems did not exist. Other PROMs like the PROMIS [11,12], SF-36 [13,14], and the MSQ [15] are powerful assessment tools in their own right, but none were created to be a digital first in this new era of telehealth-centric care delivery. Although the PROMIS has many useful short forms, the most general ones were not designed to replace the ROS in the clinical encounter.

However, these results suggest that further validation of the PSI would benefit from comparing it to a PROM with similar granularity (eg, bodily system level) even if this PROM would not be a perfect conceptual match. The PROMIS, SF-36, and MSQ are similar enough that we hope to compare these tools to the PSI in future studies to better understand the PSI as a conceptually valid yet distinctly useful tool.

### Limitations

The majority of Parsley Health members are White and female, so the study population was skewed in that direction, limiting the ecological validity of our results. Additionally, there was no randomization of PSI and SRH item presentation to address response biases. As the SRH was nested within the existing PSI, the infrastructure of the electronic health record could not support randomization. Future studies should consider randomization or A/B testing. There was also a lack of conceptually and operationally similar PROMs which we could use to validate the PSI. This is the reason that we created the PSI. In this study, we chose to compare the PSI to the SRH, a single-item questionnaire, to demonstrate convergent validity. Future studies will compare the PSI to PROMs that are similar in item length if not perfect matches in their design and intent. 

### Conclusions

This convergent validation study compared the best available questionnaire (SRH) to the PSI. Although the SRH and PSI fall under the same conceptual umbrella, they are different in their level of granularity. Further validation studies should compare the PSI to other multi-item, short-form PROMs of similar scope to continue the validation process. As telehealth will inevitably continue to grow, PROMs will be increasingly used and built as exclusively digital tools. Therefore, PROMs being used in the digital space must be researched and validated within the telehealth environment. This is a paradigm shift in the world of PROM development and validation. As this field evolves, we will need to assess what it means to validate a tool that is no longer administered to a captive audience in a physical waiting room, but rather, one that is engaged with remotely. Measures of engagement and “stickiness” will need to be considered as we build tools that can be completed anywhere and at any time of the day. These digital PROMs will need to be validated against previously validated tools while also being able to stand up to the test of our modern, all-access world.

## Appendix

Multimedia Appendix 1 Checklist for reporting results of internet e-surveys.

## Abbreviations

CHERRIES: Checklist for Reporting Results of Internet E-Surveys
e-PROM: electronic patient-reported outcomes measure
FDA: Federal Drug Agency
MSQ: Medical Symptom Toxicity Questionnaire
PROMIS: Patient-Reported Outcomes Measurement Information System
PSI: Parsley Symptom Index
ROS: review of systems
SF-36: 36-Item Short Form Health Survey
SRH: self-rated health
