# Supplementary material for: Validity of the Parsley Symptom Index—an Electronic Patient-Reported Outcomes Measure Designed for Telehealth: Prospective Cohort Study
Source: JMIR Form Res. 2022 Nov 3;6(11):e40063. doi: 10.2196/40063 (PMC9673004; doi:10.2196/40063)
Supplement: Multimedia Appendix 1 [file formative_v6i11e40063_app1.pdf]

Table S1. Checklist for reporting results of internet e-surveys.

	Description	Explanation	Section	Page Number
**Survey Design**	Describe survey design	Prospective cohort study	Methods	4
**IRB approval**	Mention whether the study has been approved by an IRB.	IRB approval/cite paper	Methods	4
**Informed consent**	Describe the informed consent process. Where were the participants told the length of time of the survey, which data were stored and where and for how long, who the investigator was, and the purpose of the study?	Exempt	Methods	4
**Data protection**	If any personal information was collected or stored, describe what mechanisms were used to protect unauthorized access.	Refer to citation 18	Methods	4
**Development and testing**	State how the survey was developed, including whether the usability and technical functionality of the electronic questionnaire had been tested before fielding the questionnaire.	Refer to citation 18	Methods	5
**Open Survey versus closed survey**	An "open survey" is a survey open for each visitor of a site, while a closed survey is only open to a sample which the investigator knows (password-protected survey).	Closed survey	Methods	5-6
**Contact mode**	Indicate whether or not the initial contact with the potential participants was made on the Internet. (Investigators may also send out questionnaires by mail and allow for Web-based data entry.)	Initial contact through membership enrollment	Methods	4-5
**Administering the survey**	How/where was the survey announced or advertised? Some examples are offline media (newspapers), or online (mailing lists – If yes, which ones?) or banner ads (Where were these banner ads posted and what did they look like?). It is important to know the wording of the announcement as it will heavily influence who chooses to participate. Ideally the survey	Not advertised. Administered as part of Parsley membership intake process	Methods	5-6

	announcement should be published as an appendix.			
**Web/e-mail**	State the type of e-survey (eg, one posted on a Web site, or one sent out through e-mail). If it is an e-mail survey, were the responses entered manually into a database, or was there an automatic method for capturing responses?	Web	Methods	5
**Context**	Describe the Web site (for mailing list/newsgroup) in which the survey was posted. What is the Web site about, who is visiting it, what are visitors normally looking for? Discuss to what degree the content of the Web site could pre-select the sample or influence the results. For example, a survey about vaccination on a anti-immunization Web site will have different results from a Web survey conducted on a government Web site	my.parsleyhealth.com websit; password protected portal for Parsley Health Members	Methods	5-6
**Mandatory/ voluntary**	Was it a mandatory survey to be filled in by every visitor who wanted to enter the Web site, or was it a voluntary survey?	Mandatory for first visit, requested and encouraged for follow ups	Methods	5
**Incentives**	Were any incentives offered (eg, monetary, prizes, or non-monetary incentives such as an offer to provide the survey results)?	No monetary incentives. Feedback of 1) doing survey 2) discussing survey with clinician 3) reviewing results over time with clinician (graph	Methods/ appendix	5
**Time/date**	In what timeframe were the data collected?	January 15, 2021 to December 15, 2021	Methods	5
**Randomization of items or questionnaires**	To prevent biases items can be randomized or alternated.	Order of PSI and SRH not randomized due to technical limitations of the system. . Could be addressed in future studies, but more likely to compare to different	Methods/ discussion	5, 9

		questionnaires.		
**Adaptive questioning**	Use adaptive questioning (certain items, or only conditionally displayed based on responses to other items) to reduce the number and complexity of the questions.	Refer to citation 18	Methods	5-6
**Number of items**	What was the number of questionnaire items per page? The number of items is an important factor for the completion rate.	1 item per page	Table 4	N/A
**Number of screens (pages)**	Over how many pages was the questionnaire distributed? The number of items is an important factor for the completion rate.	48 - 93 depending on adaptive questions/conditional responses	Table 4	N/A
**Completeness check**	It is technically possible to do consistency or completeness checks before the questionnaire is submitted. Was this done, and if "yes", how (usually JAVAScript)? An alternative is to check for completeness after the questionnaire has been submitted (and highlight mandatory items). If this has been done, it should be reported. All items should provide a non-response option such as "not applicable" or "rather not say", and selection of one response option should be enforced.	Yes. A dedicated team of software engineers (internal) performed pre go-live checks on data validation and collection	Table 4	N/A
**Review step**	State whether respondents were able to review and change their answers (eg, through a Back button or a Review step which displays a summary of the responses and asks the respondents if they are correct).	Yes. Participants have the option of using the back button to change answers.	Table 4	N/A
**Unique site visitor**	If you provide view rates or participation rates, you need to define how you determined a unique visitor. There are different techniques available, based on IP addresses or cookies or both.	Survey completed through online portal that requires registration and login for repeated visits.	Table 4	N/A
**View rate (ratio of unique survey visitors/unique site visitors)**	Requires counting unique visitors to the first page of the survey, divided by the number of unique site visitors (not page views!). It is not unusual to have view rates of less than 0.1 % if the survey is voluntary.	N/A	N/A	N/A

**Participation rate (ratio of unique visitors who agreed to participate/unique first survey page visitors)**	The number of people submitting the last questionnaire page, divided by the number of people who agreed to participate (or submitted the first survey page). This is only relevant if there is a separate "informed consent" page or if the survey goes over several pages. This is a measure for attrition. Note that "completion" can involve leaving questionnaire items blank. This is not a measure for how completely questionnaires were filled in. (If you need a measure for this, use the word "completeness rate".)	Average monthly PSI completion rate was 77% (69-83%) over the study period.	Methods	5-6
**Cookies used**	Indicate whether cookies were used to assign a unique user identifier to each client computer. If so, mention the page on which the cookie was set and read, and how long the cookie was valid. Were duplicate entries avoided by preventing users access to the survey twice; or were duplicate database entries having the same user ID eliminated before analysis? In the latter case, which entries were kept for analysis (eg, the first entry or the most recent)?	N/A	N/A	N/A
**IP check**		N/A; multi-factor authentication is required to login which is more accurate then IP (e.g., multiple patients on same network)	Table 4	N/A
**Log file analysis**		Not performed.	N/A	N/A
**Registration**		Yes, membership is required	Methods	4-5
**Handling of incomplete questionnaires**	Were only completed questionnaires analyzed? Were questionnaires which terminated early (where, for example, users did not go through all questionnaire pages) also analyzed?	PSI has to be complete to be included in the sample population per inclusion criteria.	Methods	4-6

**Questionnaires submitted with an atypical timestamp**	Some investigators may measure the time people needed to fill in a questionnaire and exclude questionnaires that were submitted too soon. Specify the timeframe that was used as a cut-off point, and describe how this point was determined.	No assessments were excluded based on time receipts. Data from the initial publication showed that 82% of patients the PSI took less than 5 minutes to complete, 14% completed the PSI between 5 and 10 minutes, and 4% took more than 10 minutes to complete the PSI. The design of 1 item per screen was used to protect against the PSI being completed too quickly, making it impossible to do the PSI in less than one minute.	Table 4	N/A
**Statistical correction**	Indicate whether any methods such as weighting of items or propensity scores have been used to adjust for the non-representative sample; if so, please describe the methods.	No item weight was used or adjusted for the non-representative sample.	Table 4	N/A
